# Radiofrequency Microporation Enhances Topical Minoxidil Delivery and Hair Regeneration in Androgenetic Alopecia

**DOI:** 10.3390/pharmaceutics18091081

**Published:** 2026-08-28

**Authors:** Na-Young Yu, Kyu-Jin Cho, Saeeun Ryu, Gyulim Kim, Jae-Woo Shin, Donghee Park, Jongho Won, Young-Kee Shin, Eun-Ah Kim, Sang-Goo Cho, Nae-Won Kang, Byung-Hoon Lee, Nam-Young Kim, Eun-Seong Kim, Dae-Duk Kim

**Affiliations:** 1 College of Pharmacy, Seoul National University, Seoul 08826, Republic of Korea; nyyu8520@snu.ac.kr (N.-Y.Y.); you7364@snu.ac.kr (S.R.); gyulim1089@snu.ac.kr (G.K.); ykeeshin@snu.ac.kr (Y.-K.S.); lee@snu.ac.kr (B.-H.L.); 2 APR Co., Ltd., Seoul 05551, Republic of Korea; kjcho@apr-in.com (K.-J.C.); zoeshin@apr-in.com (J.-W.S.); donghee.park@apr-in.com (D.P.); won.jh@apr-in.com (J.W.); 3 RF Bio Center, Kwangwoon University, Seoul 01897, Republic of Korea; eakim3037@gmail.com (E.-A.K.); nykim@kw.ac.kr (N.-Y.K.); 4 Laboratory of Molecular Pathology and Cancer Genomics, Department of Molecular Medicine and Biopharmaceutical Sciences, Graduate School of Convergence Science and Technology, Seoul National University, Seoul 08826, Republic of Korea; 5 Department of Stem Cell and Regenerative Biotechnology, School of Advanced Biotechnology, Molecular & Cellular Reprogramming Center, Institute of Advanced Regenerative Science, Konkuk University, Seoul 05029, Republic of Korea; ssangoo@konkuk.ac.kr; 6 College of Pharmacy, The Catholic University of Korea, Bucheon-si 14662, Republic of Korea; nwkang@catholic.ac.kr

**Keywords:** radiofrequency microporation, minoxidil, skin microchannels, hair regeneration, androgenetic alopecia, topical drug delivery

## Abstract

**Background/Objectives**: Androgenetic alopecia (AGA) is the most prevalent form of hair loss. Although topical minoxidil (MNX) is widely used to treat AGA, its efficacy is limited by poor penetration across the stratum corneum. This study evaluated radiofrequency (RF) microporation as a means of enhancing cutaneous MNX delivery and hair-regrowth efficacy in a dihydrotestosterone (DHT)-induced AGA mouse model. **Methods**: RF-induced skin permeabilization and barrier recovery were assessed in rats using methylene blue and rhodamine B staining. In vivo skin deposition and pharmacokinetic studies were conducted to quantify cutaneous MNX accumulation and systemic exposure. Hair-regrowth efficacy was evaluated in DHT-treated mice. **Results:** RF microporation generated transient microchannels in the stratum corneum, increased rhodamine B penetration into deeper skin layers, and allowed substantial barrier recovery within 24 h. RF pretreatment significantly increased MNX deposition in the epidermis/dermis by 2.40-fold at 1 h and 1.94-fold at 3 h compared with topical MNX alone. RF-assisted topical administration resulted in a relative bioavailability of 11.17%, compared with 4.74% for topical administration without RF, while dose-normalized systemic exposure remained substantially lower than that following oral administration. In the AGA model, RF-assisted MNX treatment significantly increased hair coverage, length, and shaft thickness. Histological analysis further showed more prominent follicular structures in the RF-assisted MNX groups. **Conclusions**: RF microporation creates transient microchannels that enhance cutaneous MNX delivery and improve hair-regrowth efficacy. Importantly, RF-assisted topical administration maintained substantially lower systemic exposure than oral administration, supporting its potential as a needle-free strategy for topical AGA therapy and further translational evaluation.

## 1. Introduction

Androgenetic alopecia (AGA) is a common hair-loss disorder characterized by progressive miniaturization of hair follicles and shortening of the anagen phase [1]. Testosterone is converted to dihydrotestosterone (DHT) by 5α-reductase, and DHT binds to androgen receptors [2]. This androgen signaling alters the expression of factors involved in hair-cycle regulation, progressively shortening the anagen phase and promoting follicular miniaturization, ultimately resulting in shorter and thinner hair shafts [3]. Topical minoxidil (MNX) and oral finasteride are currently approved by the U.S. Food and Drug Administration (FDA) for AGA treatment [4]. Oral minoxidil is also used off-label for hair loss; however, increased systemic exposure may elevate the risk of adverse effects, including hypertrichosis [5,6]. Topical MNX limits systemic absorption, but its efficacy is constrained by poor penetration across the stratum corneum [7]. In addition, the need for long-term daily application may reduce treatment adherence and contribute to discontinuation [8,9]. Therefore, strategies that enhance cutaneous MNX delivery are needed to improve the therapeutic utility of topical AGA treatment.

The stratum corneum, the outermost skin layer, is the principal physicochemical barrier to the absorption of topically applied drugs [10]. Its barrier function arises primarily from a highly ordered lamellar lipid architecture composed of ceramides, free fatty acids, and cholesterol [11]. This structure markedly restricts drug penetration into deeper skin layers [12]. For MNX, enhanced cutaneous delivery is particularly important because pharmacological activity requires sufficient concentrations in and around hair follicles, as well as in the surrounding epidermal and dermal compartments. Because hair follicles extend into the dermis, increasing MNX deposition in these regions may enhance its hair-growth-promoting effects [13].

Various chemical and physical approaches have been investigated to overcome the stratum corneum barrier. Chemical penetration enhancers can improve cutaneous drug delivery by disrupting the lipid organization of the stratum corneum [14], but some compounds may cause irritation or cytotoxicity [15,16]. Physical enhancement methods, including iontophoresis and microneedles, can also increase skin absorption. Iontophoresis uses a low electrical current to drive charged drugs or formulations into the skin from an electrode of the same polarity [17]. However, iontophoretic transport depends strongly on drug and formulation properties, including ionization state, molecular size, pH, and ionic strength [18,19]. Microneedles directly penetrate the stratum corneum to create microchannels for drug delivery. Despite their effectiveness, microneedle systems may be limited by drug-loading capacity, mechanical-strength requirements, insertion-related considerations, and the need for drug- or formulation-specific designs [20,21]. In contrast, RF microporation creates transient microchannels through localized electrical microdischarges without conventional needle insertion. This needle-free and transient approach would therefore be advantageous for topical therapies requiring repeated or long-term administration.

Radiofrequency (RF) microporation transiently increases skin permeability by creating microchannels that bypass the lipid-diffusion pathway of the stratum corneum [22]. Applying high-frequency electrical energy between a metal electrode and the skin surface causes dielectric breakdown of the intervening air gap, generating a focused discharge that ablates the superficial keratinized layer [23]. The resulting aqueous microchannels provide direct pathways for drug transport across the stratum corneum [24]. When RF parameters are appropriately controlled, ablation is confined to the superficial keratin-rich layers, preserving the viable epidermis and dermis [23,25]. This selectivity is attributed to the low thermal energy delivered per pulse and the self-limiting electrical discharge, which terminates upon direct electrode–skin contact. RF-generated microchannels, typically approximately 100 μm in diameter, facilitate drug transport while preserving the structural integrity of the underlying viable tissue [23,26]. The microchannels are transient, and skin barrier function is progressively restored within hours through natural repair processes [24]. This rapid recovery may limit prolonged systemic exposure and favor repeated application [27].

**Figure 1:**
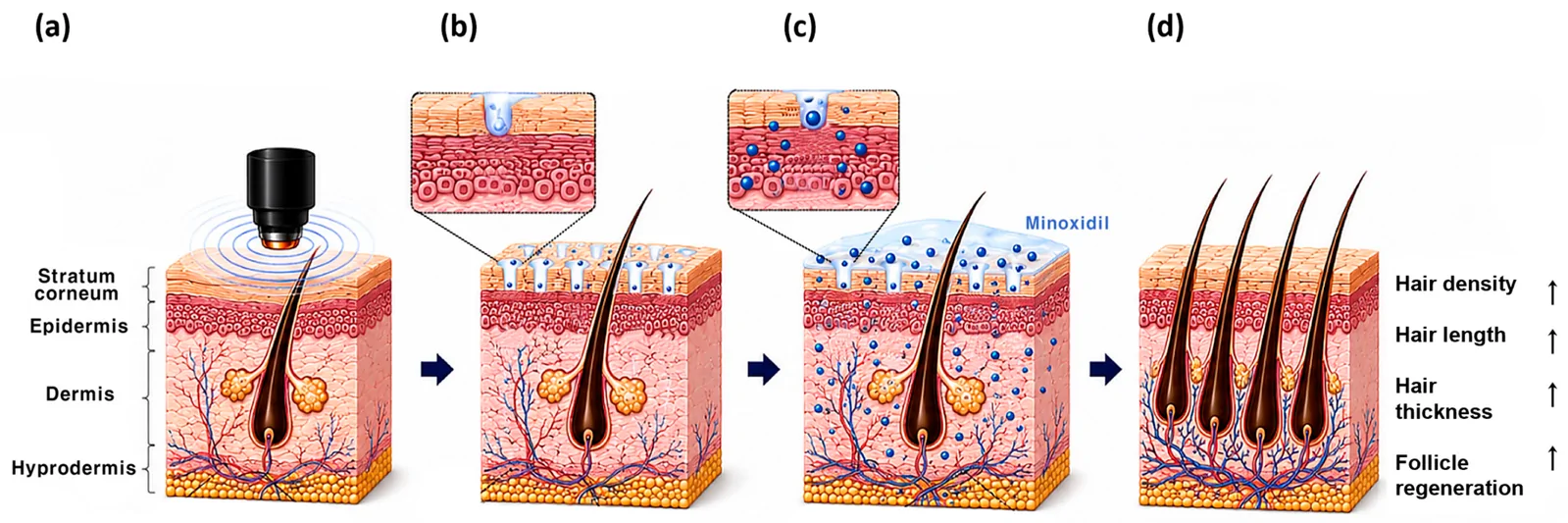
Schematic illustration of RF microporation-mediated enhancement of topical minoxidil delivery and hair regeneration in androgenetic alopecia. (**a**) RF microporation of the skin; (**b**) transient microchannel formation in the stratum corneum; (**c**) enhanced minoxidil delivery through RF-generated microchannels; and (**d**) improved hair regeneration.

Previous studies have primarily shown that RF microporation enhances the skin penetration of fluorescent dyes and model compounds [23,24,28]. However, evidence remains limited regarding whether RF microporation can enhance the cutaneous absorption of an active pharmaceutical ingredient and whether improved delivery translates into pharmacological efficacy. Therefore, this study investigated whether RF-generated skin microchannels increase the absorption and deposition of MNX and enhance its pharmacological activity in a DHT-induced AGA mouse model (Figure 1).

## 2. Materials and Methods

### 2.1. Materials

Minoxidil (MNX) and dihydrotestosterone (DHT) were purchased from Tokyo Chemical Industry Co., Ltd. (Tokyo, Japan). Commercial 5% Minoxyl topical gel and 5% Minoxyl topical solution, manufactured by Hyundai Pharm Co., Ltd. (Seoul, Republic of Korea), were purchased from a local pharmacy. High-performance liquid chromatography (HPLC)-grade methanol, acetonitrile, and water were purchased from Thermo Fisher Scientific (Pittsburgh, PA, USA). All other reagents were of analytical grade.

### 2.2. Animals

Male 8-week-old Sprague–Dawley (SD) rats were obtained from Koatech Inc. (Gyeonggi-do, Republic of Korea), and male 7-week-old C57BL/6 mice were purchased from Narabiotech Inc. (Seoul, Republic of Korea). All animal experiments were conducted in accordance with a protocol approved by the Institutional Animal Care and Use Committee of Seoul National University (approval No. SNU-250812-3-1; approval date: 22 September 2025). Animals were housed in a temperature- and humidity-controlled room under a 12 h light/12 h dark cycle at 22 ± 2 °C and 55 ± 5% relative humidity.

### 2.3. RF Microporation Device

Skin microporation was performed using a handheld unipolar radiofrequency (RF) device (Booster Pro, APR Co., Ltd., Seoul, Republic of Korea) operated in Air Shot mode, which generates transient localized electrical microdischarges across the electrode-skin air gap. These microdischarges superficially ablate the stratum corneum to form discrete micropores. (Supplementary Figure S1). Unlike conventional electroporation, which transiently perturbs lipid bilayers through electric-field-induced permeabilization, the RF microporation device physically creates microchannels by inducing dielectric breakdown in the air gap between the electrode and the skin surface. The device converts a low-voltage input to a high-voltage RF output using a step-up transformer; voltage amplification occurs through electromagnetic induction between the primary and secondary windings. A carrier frequency of 100 kHz was used to facilitate repeatable air-gap breakdown and electrical microdischarge generation under the electrode geometry and operating conditions of the device [23,29]. The carrier signal was modulated at a burst frequency of 40 Hz to produce pulsed discharges and minimize thermal accumulation within the treatment area. Five duty-cycle conditions (12%, 28%, 36%, 44%, and 48%) were evaluated. Duty cycle was calculated using the following equation:

Duty cycle (%) = (pulse duration/pulse repetition interval) × 100

Modulation of the duty cycle controlled the duration of the electrical microdischarge within each burst, thereby regulating the density and dimensions of the resulting microchannels. The device uses a spherical stainless-steel unipolar electrode to localize the electrical microdischarge to a confined area of the skin surface. As the electrode approaches the skin, dielectric breakdown of the intervening air gap initiates a localized electrical microdischarge. The resulting thermal energy selectively ablates the superficial keratinized layers of the stratum corneum, producing discrete micropores while preserving the viable epidermis and dermis. The electrical discharge is self-limiting and terminates immediately upon direct electrode–skin contact, thereby preventing excessive tissue heating and enabling reproducible microchannel generation [23]. During treatment, the RF electrode was held in close proximity to the skin surface and gently swept, brushed, or tapped across the designated treatment area while maintaining a minimal air gap sufficient for localized electrical microdischarge generation.

### 2.4. Assessment of Skin Permeabilization and Barrier Recovery

RF microporation-induced skin permeabilization was evaluated in SD rats using methylene blue staining and rhodamine B fluorescence imaging. For methylene blue staining, rats were anesthetized with Zoletil (Virbac, Carros, France), and the dorsal skin was secured beneath a Keshary–Chien diffusion chamber (1.77 cm^2^). Animals were assigned to an untreated control group or RF-treated groups. RF microporation was applied to the dorsal skin for 1 min in the RF-treated groups, whereas the control group received no RF treatment. A 0.1% methylene blue solution was then applied to the treatment area either immediately or 24 h after RF microporation. After 3 min, the rats were euthanized, and the treated skin was excised and examined using a digital microscope (Dino-Lite, Taipei, Taiwan).

To further assess skin permeabilization and barrier recovery, a 0.01% rhodamine B solution was applied either immediately or 24 h after RF treatment. After 1 h, the skin surface was gently cleaned with methanol and double-distilled water (DDW), and the skin samples were collected. Skin surfaces were examined using a digital microscope. The samples were then cryosectioned, and fluorescence images were acquired using an Axio Scan slide scanner (Zeiss, Oberkochen, Germany). All results were compared with those from untreated rats.

### 2.5. In Vivo Skin Deposition Study

In vivo skin deposition of MNX was evaluated in SD rats using a previously described method with minor modifications [30]. Dorsal hair was removed with an electric clipper and depilatory cream 24 h before the experiment. The rats were anesthetized, and a Keshary–Chien diffusion cell (1.77 cm^2^ surface area) was secured to the dorsal skin. Animals were assigned to a control group or an RF microporation group (n = 3 per group). In the RF group, RF microporation was applied for 1 min to the dorsal skin area defined by the diffusion cell, whereas the control group received no RF treatment. Subsequently, 1.0 mL of 0.5% MNX solution, prepared by diluting 5% Minoxyl solution with DDW, was added to the donor chamber. At 1 or 3 h after application, the rats were euthanized, and the treated skin was washed with methanol and DDW. To separate the stratum corneum from the underlying skin, cellophane adhesive tape (CuDerm Corporation, Dallas, TX, USA) was applied to the treated area three consecutive times. Each tape strip was collected in a 2 mL tube containing methanol. The remaining epidermal and dermal tissues were chopped with scissors and ground into fine powder using a mortar and pestle in the presence of liquid nitrogen. The powder was transferred to a 2 mL tube containing methanol. MNX was extracted from the tape and skin tissues by vortex mixing for 3 h, followed by centrifugation at 16,000× *g* for 5 min. MNX in the stratum corneum and combined epidermal/dermal tissues was quantified using liquid chromatography-tandem mass spectrometry (LC-MS/MS).

### 2.6. In Vivo Pharmacokinetic Study

A pharmacokinetic study of MNX was conducted in SD rats. After hair removal with depilatory cream, the rats were anesthetized with Zoletil (50 mg/kg, intramuscularly). The femoral artery was catheterized using polyethylene tubing (Intramedic PE-50^®^; Becton Dickinson Diagnostics, NJ, USA). MNX was administered orally at 1.0 mg/kg or topically at 10 mg/kg. The oral group received an MNX solution in 5% ethanol/normal saline. In the topical groups, 5% Minoxyl gel was applied to a 2 × 2 cm^2^ area of abdominal skin. In the RF-treated topical group, RF microporation was applied to the treatment area for 1 min immediately before MNX gel application, whereas the topical control group received no RF treatment. Blood samples (250 μL) were collected through the femoral artery catheter at 5, 15, 30, 60, 120, 240, and 480 min after MNX administration. Samples were centrifuged at 16,000× *g* for 5 min at 4 °C. Plasma was vortex-mixed for 5 min with acetonitrile (ACN) containing carbamazepine (100 ng/mL) as the internal standard and then centrifuged again at 16,000× *g* for 5 min. Plasma MNX concentrations were quantified using LC-MS/MS. Pharmacokinetic parameters were estimated by noncompartmental analysis using WinNonlin software (version 5.0.1; Pharsight Co., Mountain View, CA, USA). The following parameters were derived from the plasma concentration-time data: maximum plasma concentration (C_max_), time to C_max_ (T_max_), elimination half-life (t_1/2_), area under the plasma concentration-time curve from time zero to the last quantifiable time point (AUC_last_), and area under the plasma concentration-time curve from time zero to infinity (AUC_inf_).

### 2.7. LC-MS/MS Analysis of MNX

MNX concentrations in rat plasma and skin samples were quantified using LC-MS/MS. The mobile phase consisted of methanol/DDW (90:10, *v*/*v*) containing 0.1% formic acid and was delivered at 0.4 mL/min. Chromatographic separation was performed on a C_18_ analytical column (Poroshell 120, 50 mm × 4.6 mm, 2.7 μm; Agilent Technologies, Santa Clara, CA, USA). The LC-MS/MS system comprised an Agilent 1260 Infinity HPLC system coupled to an Agilent 6430 Triple Quad LC-MS system (Agilent Technologies). Detection was performed in multiple reaction monitoring (MRM) mode using positive electrospray ionization (ESI). The gas temperature, gas flow rate, nebulizer pressure, and capillary voltage were 300 °C, 11 L/min, 15 psi, and 4500 V, respectively. The precursor-to-product ion transitions, fragmentor voltages, and collision energies were *m*/*z* 210.1 → 164.1, 105 V, and 17 eV for MNX and *m*/*z* 237.1 → 194.1, 140 V, and 19 eV for carbamazepine, respectively. Data were processed using MassHunter Workstation Quantitative Analysis software (version B.05.00; Agilent Technologies).

### 2.8. In Vivo Efficacy Study in a DHT-Induced AGA Mouse Model

An in vivo efficacy study was conducted in male C57BL/6 mice with DHT-induced AGA. Dorsal hair was shaved with an electric clipper and removed with depilatory cream. After 24 h, the mice were randomly assigned to five groups (n = 5 per group): DHT, MNX (L), RF + MNX (L), MNX (H), and RF + MNX (H).

A DHT-induced AGA mouse model was established using previously reported methods [31,32]. Mice in all groups were anesthetized with isoflurane and received a subcutaneous injection of DHT (30 mg/kg in corn oil) once daily for 21 consecutive days. The DHT group served as the disease control group without topical MNX treatment nor RF microporation. Minoxyl gel containing 5% MNX was applied once daily to the depilated dorsal skin. The MNX (L) and MNX (H) groups received 60 and 100 μL of Minoxyl gel, respectively. The RF + MNX (L) and RF + MNX (H) groups underwent RF microporation for 1 min at a 12% duty cycle once daily for 21 consecutive days, immediately before topical Minoxyl gel application.

Hair growth on the dorsal skin was photographed on days 1, 7, 14, and 21, and hair growth and skin pigmentation were monitored throughout the study. Because changes in murine skin pigmentation are closely associated with hair-cycle progression, they were used as a noninvasive indicator of hair-cycle stage [33,34]. Skin-color scores were assigned as follows: 1, pink with no hair growth; 2, pinkish white; 3, white; 4, grayish white; 5, gray; and 6, dark gray with mature hair regrowth [35]. Hair-covered area was quantified as a percentage of the total depilated dorsal area. On day 21, newly grown hairs were collected from the dorsal skin of each mouse. Hair samples were imaged using a digital microscope (Dino-Lite, Taipei, Taiwan), and hair length was measured using ImageJ software (v. 1.54d, National Institutes of Health, Bethesda, MD, USA). Hair-shaft thickness was measured from scanning electron microscopy (SEM) images acquired with a JCM-6000 microscope (JEOL Co., Ltd., Tokyo, Japan).

### 2.9. Histological Evaluation of Skin Samples

H&E-stained sections were evaluated qualitatively for treatment-associated differences in hair follicle morphology. On day 21, the mice were euthanized, and dorsal skin samples were collected for histological evaluation. The samples were mounted on rigid cards and fixed in 4% paraformaldehyde. The tissues were dehydrated, paraffin-embedded, sectioned, and stained with hematoxylin and eosin (H&E). Sections were imaged using an Axio Scan3 7-slide scanner (Zeiss, Oberkochen, Germany).

### 2.10. Statistical Analysis

Data are presented as the mean ± standard deviation (SD). Statistical analyses were performed using Student’s *t*-test or one-way analysis of variance (ANOVA), followed by Tukey’s post hoc multiple-comparison test, in GraphPad Prism version 9.0.2 (GraphPad Software, San Diego, CA, USA). A value of *p* < 0.05 was considered statistically significant.

## 3. Results and Discussion

### 3.1. Skin Permeabilization and Barrier Recovery After RF Microporation

To identify an appropriate RF microporation condition, duty cycles of 12%, 28%, 36%, 44%, and 48% were evaluated at a carrier frequency of 100 kHz and a burst frequency of 40 Hz. Methylene blue staining revealed a duty-cycle-dependent increase in micropore formation and dye penetration (Supplementary Figure S1). Increasing the duty cycle prolonged the cumulative discharge duration and increased thermal energy deposition, resulting in more extensive disruption of the stratum corneum and microchannel formation. However, an excessively high duty cycle may be unsuitable for repeated topical application because excessive barrier disruption could increase systemic exposure and delay stratum corneum recovery [24]. Among the conditions tested, a 12% duty cycle produced visible micropores with the least extensive skin disruption. Therefore, this condition was used in subsequent studies.

**Figure 2:**
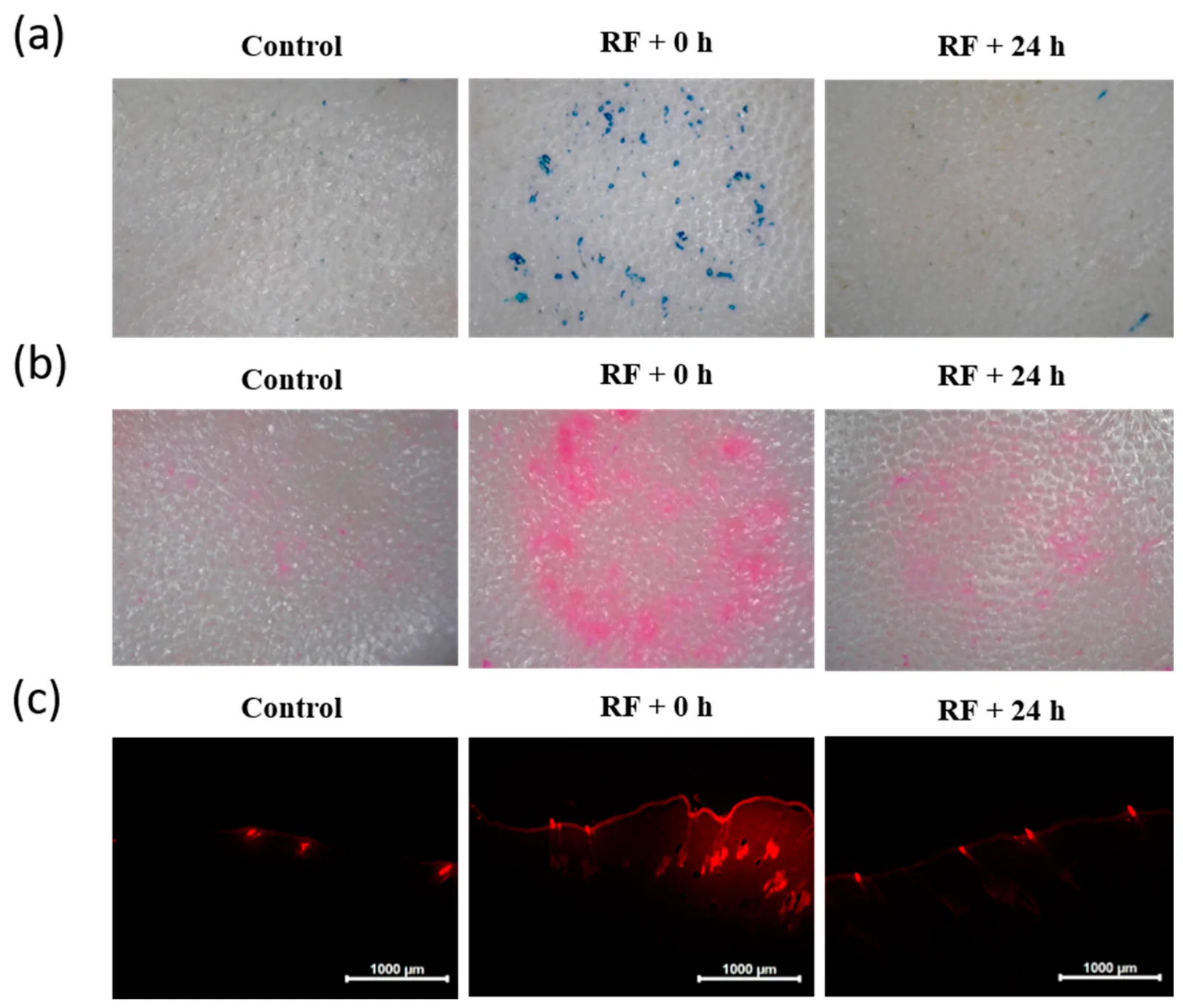
RF microporation generates transient microchannels and enhances skin permeabilization. (**a**) Methylene blue staining of rat skin without RF treatment, immediately after RF microporation, and 24 h after RF treatment. (**b**) Surface rhodamine B staining showing enhanced dye uptake after RF microporation. (**c**) Fluorescence images of rhodamine B in skin cross-sections, showing enhanced penetration into deeper layers through RF-generated microchannels and recovery of barrier function after 24 h.

To visualize RF-induced skin permeabilization, a 0.1% methylene blue solution was applied to rat skin with or without RF microporation pretreatment. As shown in Figure 2a, untreated skin exhibited minimal staining, whereas RF-treated skin showed distinct blue spots, confirming microchannel formation at the skin surface. Only weak staining was observed when the dye was applied 24 h after RF treatment, indicating that the RF-generated microchannels were transient and that the skin barrier had largely recovered. H&E-stained sections also showed pore-like disruptions in the stratum corneum after RF microporation, with no apparent structural damage to deeper skin layers (Supplementary Figure S2).

To determine whether the microchannels functionally enhanced skin permeation and whether barrier function recovered after RF treatment, rhodamine B penetration was examined in skin cross-sections. In untreated control skin, fluorescence was weak and largely confined to the surface, indicating limited penetration through the intact stratum corneum. When rhodamine B was applied immediately after RF microporation, intense fluorescence was observed in the stratum corneum and deeper skin layers, indicating enhanced penetration through RF-generated microchannels (Figure 2b,c). By contrast, fluorescence was markedly reduced when rhodamine B was applied 24 h after RF treatment. Consistent with these findings, previous studies have demonstrated gradual closure of RF-generated microchannels and restoration of barrier function following RF microporation [27]. This recovery likely reflects restoration of the disrupted stratum corneum architecture as part of the natural skin repair process, including lipid reorganization and keratinocyte-mediated repair.

These findings indicate that RF microporation transiently increased skin permeability and that the microchannels had largely resealed within 24 h as barrier function recovered. This reversible permeabilization supports the use of RF microporation as a minimally invasive approach for enhancing cutaneous drug delivery.

### 3.2. In Vivo Skin Deposition Study of Minoxidil

**Figure 3:**
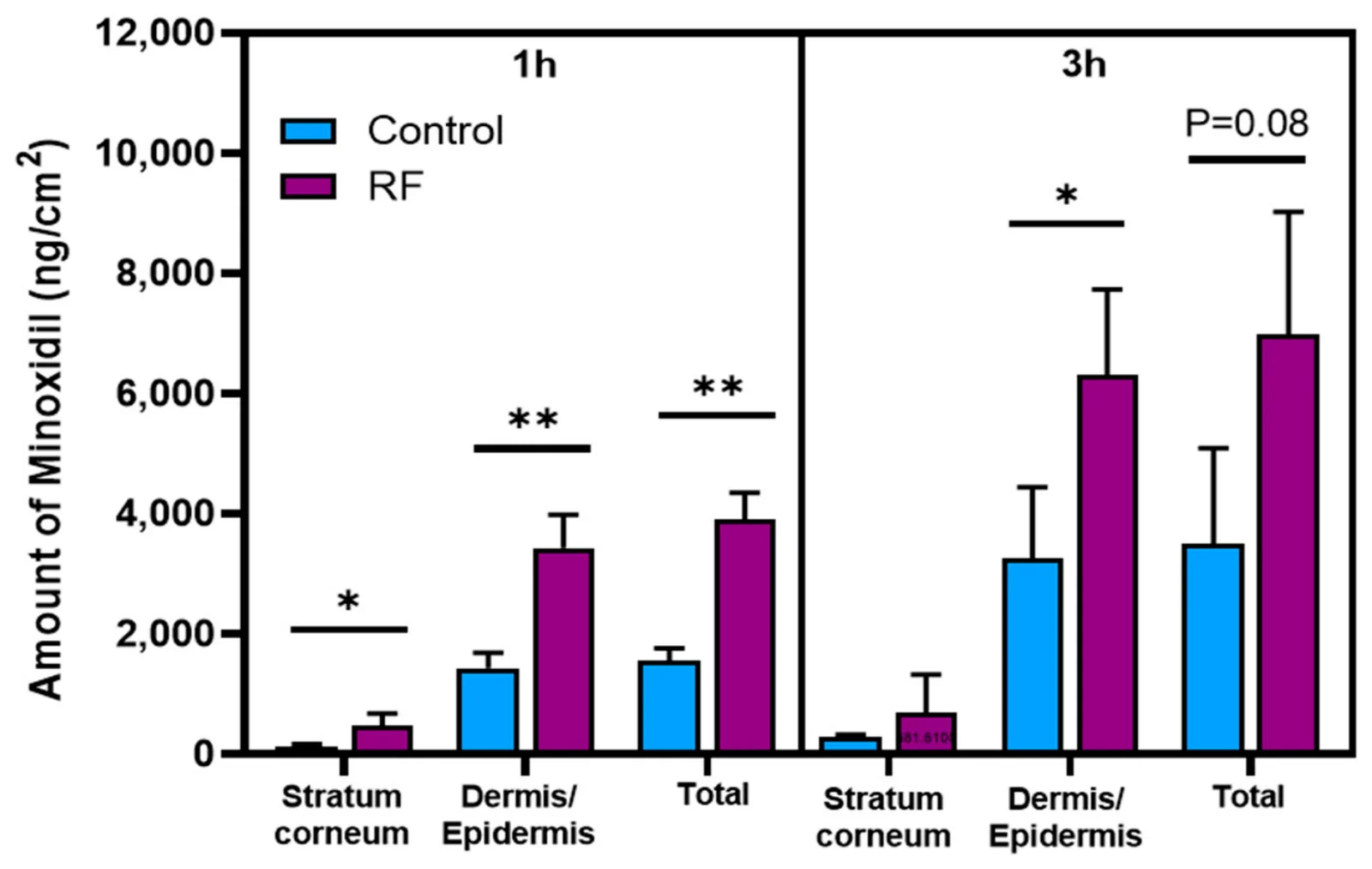
In vivo cumulative deposition of MNX in the stratum corneum and epidermis/dermis at 1 and 3 h after topical application, with or without RF microporation pretreatment. Data are presented as the mean ± SD (n = 3). * *p* < 0.05 and ** *p* < 0.01.

Figure 3 shows MNX deposition in SD rat skin at 1 and 3 h after topical application with or without RF microporation pretreatment. Minoxyl solution rather than the gel formulation was used to avoid residual gel on the skin surface after washing, which could interfere with quantification. RF pretreatment significantly increased MNX deposition in the epidermis/dermis at both 1 and 3 h relative to topical application without RF. By contrast, MNX deposition in the stratum corneum did not differ significantly between groups at 3 h. Following RF microporation, topically applied MNX is expected to penetrate through the newly formed microchannels, thereby partially bypassing the stratum corneum barrier. The significantly increased MNX deposition in the epidermis/dermis after RF pretreatment is consistent with enhanced transport across the stratum corneum through these microchannels. Although RF-generated microchannels are transient and gradually reseal, the greater MNX deposition at 3 h may reflect increased initial drug loading immediately after RF treatment. Once MNX traverses the disrupted stratum corneum, it may be retained within the skin and subsequently redistributed to deeper epidermal and dermal layers, even as barrier structure recovers. Thus, the difference in deposition at 3 h does not necessarily indicate that the microchannels remained fully open throughout the experiment; rather, it likely reflects cumulative MNX retention following early RF-mediated permeabilization.

### 3.3. In Vivo Pharmacokinetic Study of Minoxidil

**Figure 4:**
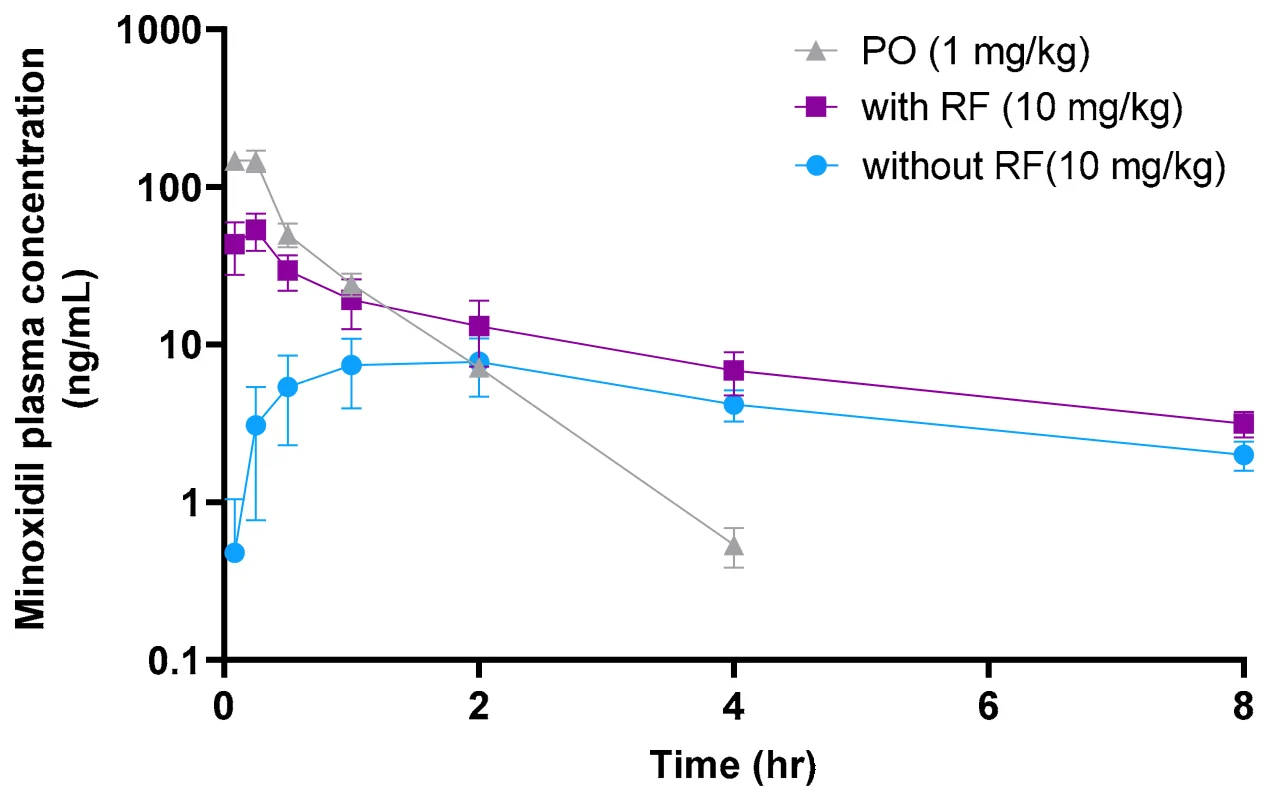
Plasma concentration-time profiles of MNX after oral administration (1.0 mg/kg) or topical administration (10 mg/kg) with or without RF microporation pretreatment in rats. Data are presented as the mean ± SD (n = 4).

The plasma concentration-time profiles of MNX after oral administration (1 mg/kg) and topical administration (10 mg/kg), with or without RF microporation pretreatment, are shown in Figure 4; the corresponding pharmacokinetic parameters are summarized in Table 1. Because excessive systemic MNX exposure can cause adverse effects such as hypertrichosis, systemic exposure after RF-assisted topical administration was evaluated. In the topical groups, the MNX gel remained on the skin throughout the sampling period, allowing continuous contact with the application site; systemic exposure may therefore reflect ongoing absorption from residual formulation. The terminal half-life (t_1/2)_ was 0.54 h after oral administration and 3.38 and 3.90 h after topical administration without and with RF microporation, respectively. The longer apparent terminal half-life after topical administration may therefore reflect prolonged absorption from the application site. After dose normalization, C_max_/dose and AUC_inf_/dose were substantially higher after oral administration than after topical administration with or without RF. Relative to topical application alone, RF pretreatment increased systemic MNX exposure, as reflected by a higher AUC_inf_, a 6.14-fold higher C_max_, and a decrease in T_max_ from 1.75 to 0.25 h, consistent with more rapid absorption through microporated skin. Nevertheless, dose-normalized relative bioavailability remained low: 4.74% without RF and 11.17% with RF. Thus, although RF microporation enhanced transdermal MNX absorption, dose-normalized C_max_ and AUC_inf_ remained substantially lower than those after oral administration. These findings indicate that RF-assisted topical delivery increases cutaneous and systemic absorption while maintaining dose-normalized systemic exposure well below that observed after oral dosing. This local-systemic exposure balance represents an advantage of RF-assisted topical MNX delivery, where enhanced local drug delivery with limited systemic exposure is desirable.

### 3.4. RF Microporation-Enhanced Hair Regrowth

We first examined whether RF-assisted MNX treatment enhanced hair regrowth in depilated healthy C57BL/6 mice (Supplementary Figure S3). Mice were assigned to four groups: untreated control, RF microporation alone, MNX alone, and RF microporation plus MNX (RF + MNX). The untreated control group received no treatment, and the RF-only group received RF microporation for 1 min once daily. The MNX-only group received 100 μL of 5% Minoxyl gel once daily, whereas the RF + MNX group underwent RF microporation for 1 min immediately before daily application of 100 μL of 5% Minoxyl gel. Hair regrowth was monitored by photographing the dorsal skin on days 0, 7, 12, and 15. No visible hair growth was observed in any group on day 7; however, clear differences in dorsal skin pigmentation emerged. The untreated control and RF-only groups retained pale-pink skin, indicating that most follicles remained in telogen. By contrast, the MNX-treated groups showed earlier pigmentation changes associated with anagen initiation. By day 12, both MNX-treated groups exhibited markedly greater grayish pigmentation than the control and RF-only groups. By day 15, visible hair growth was evident in both MNX-treated groups, and the RF + MNX group showed greater coverage of the depilated area than the MNX-only group. RF microporation alone did not significantly alter skin pigmentation or accelerate hair growth relative to the untreated control. These findings indicate that RF microporation alone did not directly stimulate hair growth under the conditions tested; rather, the enhanced regrowth after RF pretreatment was likely attributable to improved MNX delivery through RF-generated microchannels.

**Figure 5:**
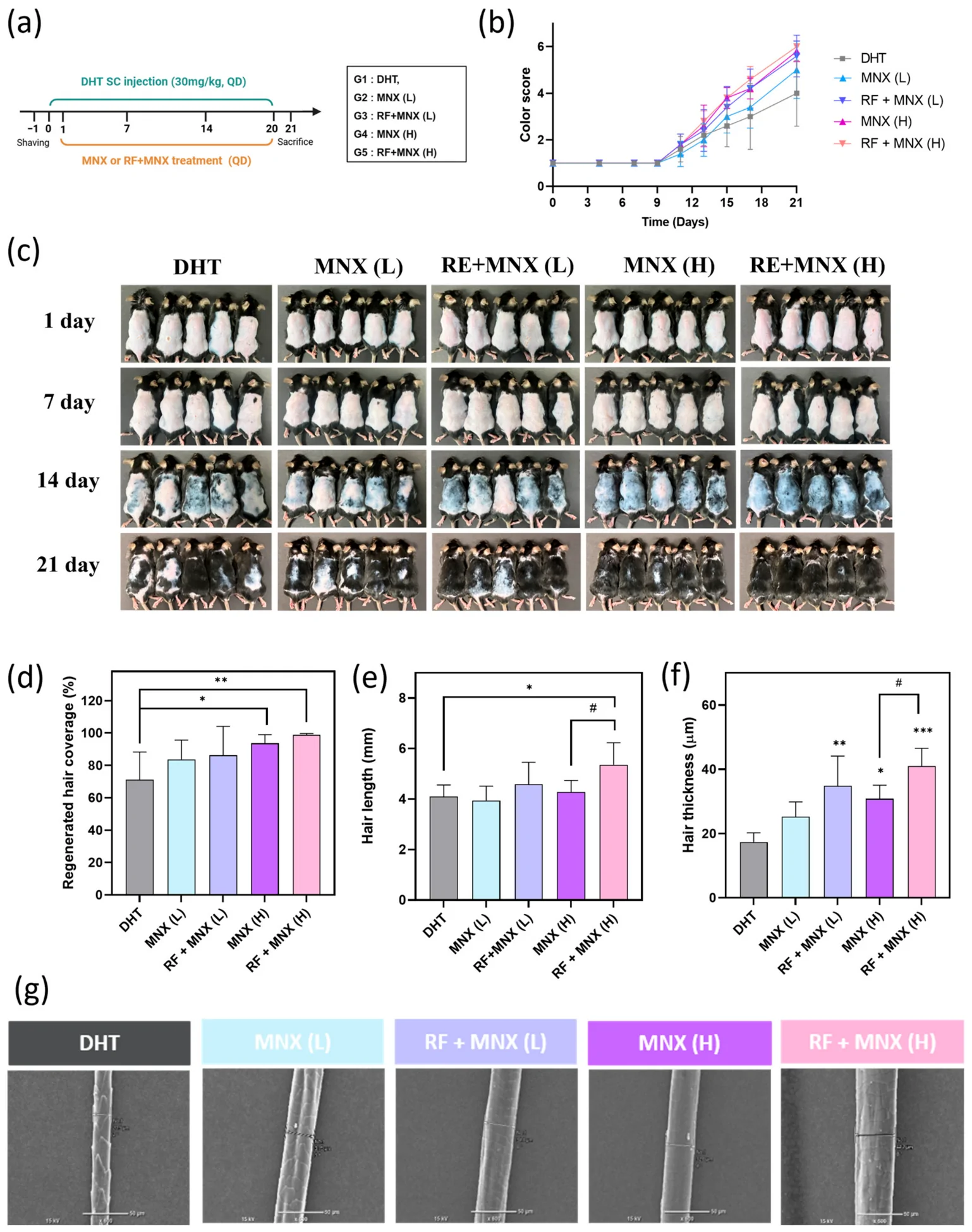
RF microporation enhances hair regeneration in a DHT-induced androgenetic alopecia (AGA) mouse model. (**a**) Experimental design and treatment schedule. (**b**) Hair-growth scores based on dorsal skin pigmentation associated with anagen induction. (**c**) Representative photographs of mice in each treatment group on days 1, 7, 14, and 21. (**d**) Hair-covered area (%) on day 21. (**e**) Length and (**f**) shaft thickness of newly regenerated hair collected from the dorsal skin on day 21. (**g**) Representative scanning electron microscopy (SEM) images of regenerated hair shafts. Scale bar = 50 μm. RF-assisted MNX treatment enhanced hair regeneration compared with MNX treatment alone. Data are presented as the mean ± SD (n = 5). * *p* < 0.05, ** *p* < 0.01, and *** *p* < 0.001 versus the DHT-induced AGA model group; # *p* < 0.05 versus the corresponding MNX-treated group.

To further assess the pharmacological relevance of RF-assisted MNX treatment, a DHT-induced AGA mouse model was treated for 21 days (Figure 5a). Dorsal skin pigmentation was monitored as an indicator of hair-cycle progression because pigmentation changes are closely associated with anagen initiation and melanogenesis in growing follicles. As shown in Figure 5b,c, all groups initially had pink dorsal skin, indicating that most follicles were in the resting phase. Differences among groups became increasingly apparent during treatment. By day 14, all mice in the RF + MNX (H) group exhibited grayish dorsal skin, suggesting accelerated entry into anagen. By day 21, hair regrowth differed markedly among groups. The DHT model group showed incomplete recovery, with hair coverage of 71.19 ± 17.07% and remaining large depilated areas. Hair regrowth was less pronounced in the MNX (L) group than in the MNX (H) group. Notably, all mice in the RF + MNX (H) group showed nearly complete coverage of the depilated area. Quantitative analysis showed that hair coverage was 93.60 ± 5.30% in the MNX (H) group and 98.72 ± 0.97% in the RF + MNX (H) group (Figure 5d). These results demonstrate that RF pretreatment further increased hair coverage compared with MNX (H) treatment alone. In addition, hair length and shaft thickness were significantly greater in the RF + MNX (H) group than in the corresponding MNX (H) group (Figure 5e–g). Collectively, these findings demonstrate that RF microporation enhanced the hair-growth-promoting efficacy of topical MNX. The superior outcomes in the RF + MNX groups suggest that RF-generated microchannels increased cutaneous MNX delivery, thereby enhancing its pharmacological activity and promoting hair regeneration in the DHT-induced AGA model.

### 3.5. Histological Evaluation

**Figure 6:**
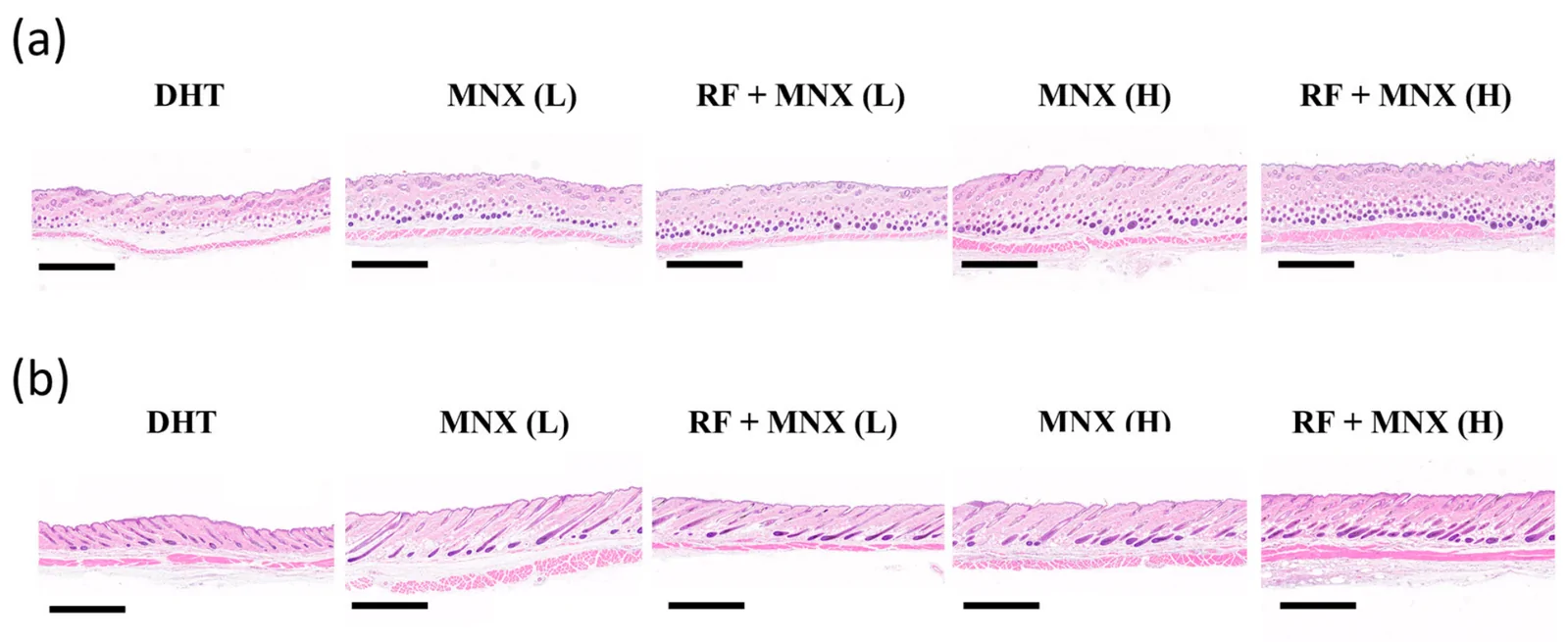
Histological analysis of hair follicle regeneration in the dorsal skin of AGA mice on day 21. Representative H&E-stained (**a**) transverse and (**b**) longitudinal sections of dorsal skin collected at the end of treatment. Scale bar = 1000 μm.

After 21 days of treatment, H&E-stained skin sections were examined for histological changes in hair follicles. The DHT model group showed relatively small, poorly developed follicular structures. MNX-treated groups showed more developed follicular morphology, whereas the RF + MNX (H) group exhibited the most prominent follicular structures among the treatment groups (Figure 6). These qualitative histological observations were consistent with the macroscopic hair-growth findings.

## 4. Conclusions

RF microporation generated transient microchannels in the stratum corneum and enhanced cutaneous MNX delivery, resulting in improved hair-regrowth responses in the DHT-induced AGA model. Importantly, although RF pretreatment increased MNX absorption compared with topical administration alone, dose-normalized systemic exposure remained substantially lower than that following oral administration. Because RF microporation can be applied as a brief pretreatment before administering an existing topical formulation, it may provide a practical needle-free strategy to improve topical MNX delivery without requiring formulation modification. However, the present findings were obtained in rodent models, and the safety and tolerability of repeated RF application, as well as its translation to human scalp skin, remain to be established. Further studies are needed to optimize RF treatment conditions, evaluate the safety and tolerability of repeated application, and confirm the efficacy of this approach in human-relevant models and clinical settings.

## Figures and Tables

**Table 1:** Noncompartmental pharmacokinetic parameters of MNX after oral administration at 1.0 mg/kg or topical administration at 10 mg/kg in rats.

Parameters	PO	Topical Without RF	Topical with RF
t_1/2_ (h)	0.54 ± 0.05	3.38 ± 0.81	3.9 ± 1.33
T_max_ (h)	0.17 ± 0.1	1.75 ± 0.5	0.25
C_max_ (ng/mL)	159.09 ± 18.82	8.65 ± 3.6	53.4 ± 14.09
C_max_/dose (ng/mL)	159.09 ± 18.82	0.87 ± 0.36	5.34 ± 1.41
AUC_inf_ (ng·h/mL)	97.54 ± 3.24	46.25 ± 11.95	108.92 ± 27.42
AUC_inf_/dose (ng·h/mL)	97.54 ± 3.24	4.62 ± 1.2	10.89 ± 2.74
Relative bioavailability (%)	100	4.74	11.17

Data are presented as the mean ± SD (n = 4).

## Data Availability

The data presented in this study are available on request from the corresponding author.

## Supplementary Materials

The following supporting information can be downloaded at: https://www.mdpi.com/article/10.3390/pharmaceutics18091081/s1, Figure S1: Visualization of microchannels generated by radiofrequency (RF) microporation at different duty cycles. (a) RF microporation device; (b) operating parameters used to optimize the RF microporation conditions; and (c) representative images of dorsal skin stained with 0.01% methylene blue without RF pretreatment (control) and after RF microporation at levels 1–5. Figure S2: Representative hematoxylin and eosin (H&E)-stained sections of untreated skin (control) and skin subjected to radiofrequency (RF) microporation for 1 min (bar: 100 μm). The opening width of a representative RF-generated micropore was approximately 77.8 μm. Figure S3: Hair regrowth in depilated healthy mice following minoxidil (MNX) treatment with or without radiofrequency (RF) microporation pretreatment. (a) Skin-color scoring criteria used to evaluate hair regrowth; (b) hair-growth scores based on changes in dorsal skin color; and (c) representative photographs of the dorsal skin of healthy mice on days 0, 7, 12, and 15 after treatment.
