# Supplementary material for: Radiofrequency Microporation Enhances Topical Minoxidil Delivery and Hair Regeneration in Androgenetic Alopecia
Source: Pharmaceutics. 2026 Aug 28;18(9):1081. doi: 10.3390/pharmaceutics18091081 (PMC13609981; doi:10.3390/pharmaceutics18091081)
Supplement: Supplementary file 1 [file pharmaceutics-18-01081-s001.zip › pharmaceutics-4502147-supplementary.pdf]

Supplementary Materials

# Radiofrequency Microporation Enhances Topical Minoxidil Delivery and Hair Regeneration in Androgenetic Alopecia

**Na-Young Yu [1], Kyu-Jin Cho [2,3], Saeeun Ryu [1], Gyulim Kim [1], Jae-Woo Shin [2], Donghee Park [2], Jongho Won [2], Young-Kee Shin [1, 4], Eun-Ah Kim [3], Sang-Goo Cho [5], Nae-Won Kang [6], Byung-Hoon Lee [1], Nam-Young Kim [3, 5], Eun-Seong Kim [4, *] and Dae-Duk Kim [1, *]**

[1] College of Pharmacy, Seoul National University, Seoul 08826, Republic of Korea; nyyu8520@snu.ac.kr (N.-Y.Y.); you7364@snu.ac.kr (S.R.); gyulim1089@snu.ac.kr (G.K.); ykeeshin@snu.ac.kr (Y.-K.S.); lee@snu.ac.kr (B.-H.L.)

[2] APR Co., Ltd., Seoul 05551, Republic of Korea; kjcho@apr-in.com (K.-J.C.); zoeshin@apr-in.com (J.-W.S.); donghee.park@apr-in.com(D.P.); won.jh@apr-in.com (J.W.)

[3] RF Bio Center, Kwangwoon University, Seoul 01897, Republic of Korea; eakim3037@gmail.com (E.-A.K.); nykim@kw.ac.kr (N.-Y.K.)

[4] Laboratory of Molecular Pathology and Cancer Genomics, Department of Molecular Medicine and Biopharmaceutical Sciences, Graduate School of Convergence Science and Technology, Seoul National University, Seoul, 08826, Republic of Korea;

[5] Department of Stem Cell and Regenerative Biotechnology, School of Advanced Biotechnology, Molecular & Cellular Reprogramming Center, Institute of Advanced Regenerative Science, Konkuk University, Seoul, 05029, Republic of Korea; ssangoo@konkuk.ac.kr (S.-G. C.)

[6] College of Pharmacy, The Catholic University of Korea, Bucheon-si, 14662, Republic of Korea; nwkang@catholic.ac.kr (N.-W.K.)

* Correspondence: 3037eskim@gmail.com (E.-S.K.); ddkim@snu.ac.kr (D.-D.K.); Tel.: +8210-7137-5071(E.-S.K.); +82-880-7870 (D.-D.K.)

(a)

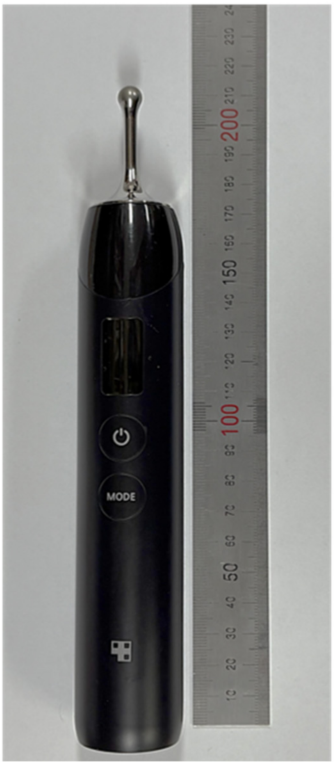


(b)

| Level | Duty Cycle (%) | Carrier Frequency (kHz) | Burst Frequency (Hz) | Electrode Material |
|---|---|---|---|---|
| 1 | 12 | 100 | 40 | Stainless Steel |
| 2 | 28 | 100 | 40 | Stainless Steel |
| 3 | 36 | 100 | 40 | Stainless Steel |
| 4 | 44 | 100 | 40 | Stainless Steel |
| 5 | 48 | 100 | 40 | Stainless Steel |

(c)

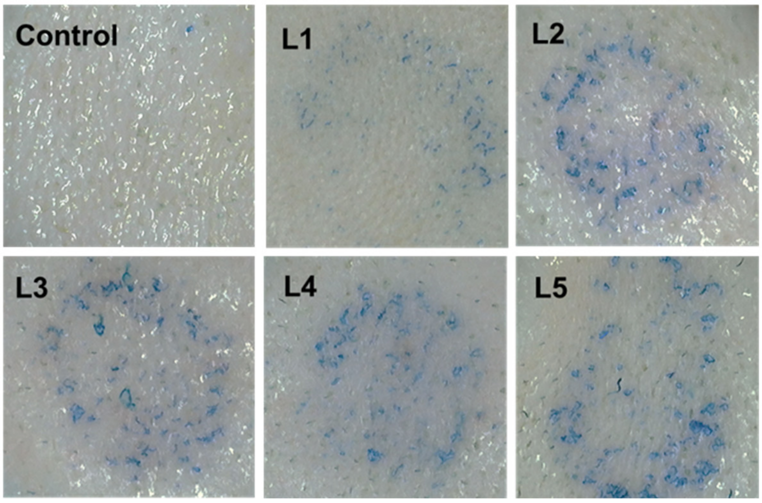


**Figure S1.** Visualization of microchannels generated by radiofrequency (RF) microporation at different duty cycles. (a) RF microporation device; (b) operating parameters used to optimize the RF microporation conditions; and (c) representative images of dorsal skin stained with 0.01% methylene blue without RF pretreatment (control) and after RF microporation at levels 1-5.

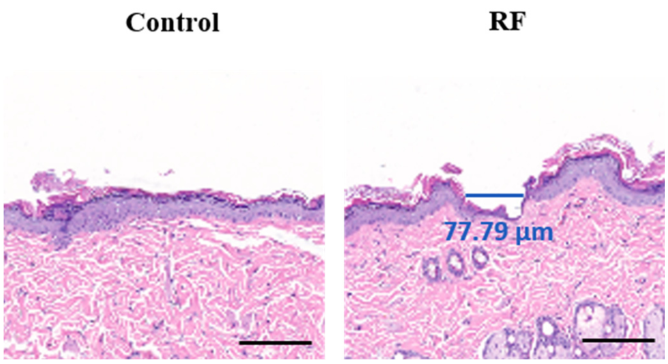


**Figure S2.** Representative hematoxylin and eosin (H&E)-stained sections of untreated skin (control) and skin subjected to radiofrequency (RF) microporation for 1 min (bar: 100 µm). The opening width of a representative RF-generated micropore was approximately 77.8 µm.

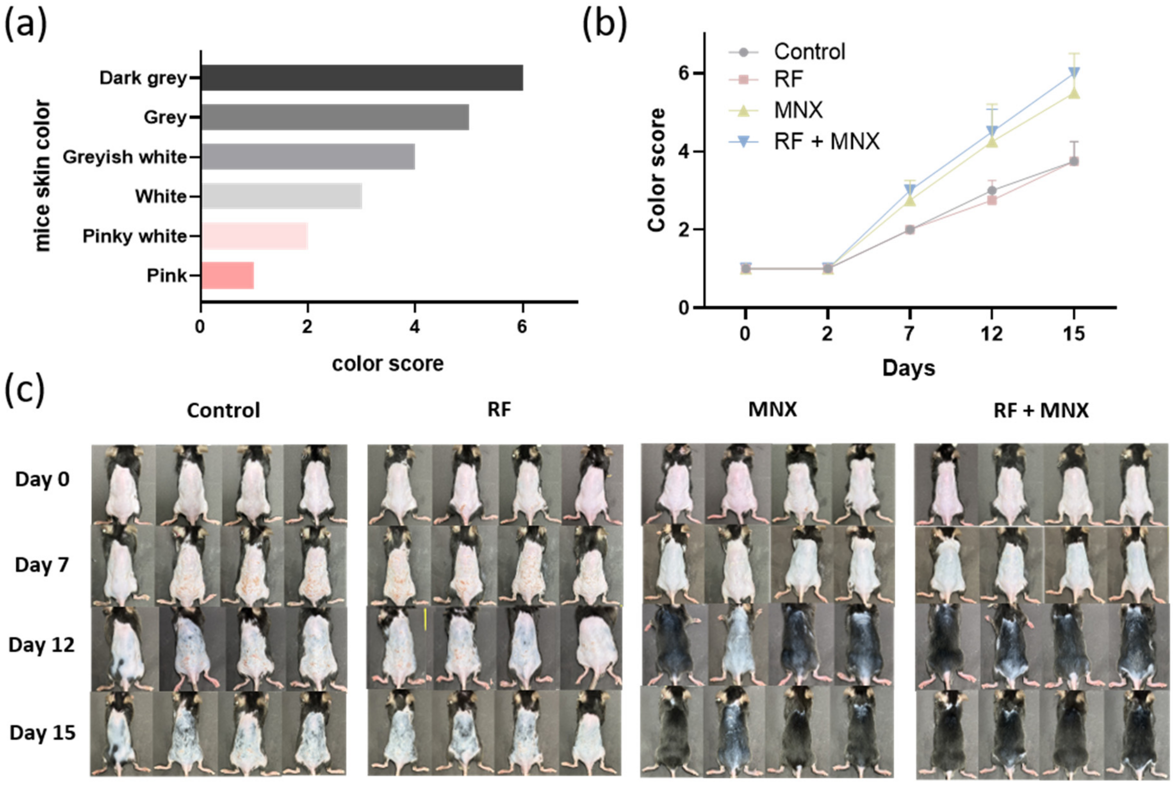


**Figure S3.** Hair regrowth in depilated healthy mice following minoxidil (MNX) treatment with or without radiofrequency (RF) microporation pretreatment. (a) Skin-color scoring criteria used to evaluate hair regrowth; (b) hair-growth scores based on changes in dorsal skin color; and (c) representative photographs of the dorsal skin of healthy mice on days 0, 7, 12, and 15 after treatment.
